# Understanding COVID-19 vaccine hesitancy in Malaysia: Public perception, knowledge, and acceptance

**DOI:** 10.1371/journal.pone.0284973

**Published:** 2023-04-27

**Authors:** Nurul Azmawati Mohamed, Hana Maizuliana Solehan, Mohd Dzulkhairi Mohd Rani, Muslimah Ithnin, Mahalecthumy Arujanan

**Affiliations:** 1 Faculty of Medicine and Health Sciences, Universiti Sains Islam Malaysia, Negeri Sembilan, Malaysia; 2 Malaysian Biotechnology Information Centre, Malaysia; 3 The Petri Dish, Malaysia; Universitat Luzern, SWITZERLAND

## Abstract

**Background:**

Vaccine hesitancy has been around since the introduction of smallpox vaccine. Vaccine hesitancy has become more intense due to the rise of vaccine information in social media and mass adult vaccination during COVID-19 pandemic. This study investigated knowledge, perception, and reasons for rejection of the COVID-19 vaccine among Malaysian adults who refused to get free COVID-19 vaccination.

**Methods:**

An online cross-sectional survey using an embedded mixed-method study [QUAN(quali)] was conducted among Malaysian adults. The quantitative section consisted of a 49-item questionnaire, whereas the qualitative sections consisted of two open-ended questions (1) "Please state your reason why you have not registered or have no intention to register at all for COVID-19 vaccines?" and (2) "Please tell us if you have any suggestions for improvement about COVID-19 vaccine delivery". Data from respondents who were not willing to get vaccination were extracted from the overall data and further analyzed in this paper.

**Result:**

Sixty-one adults completed the online open-ended survey with a mean age of 34.28 years (SD = 10.30). Among factors that influenced them to get vaccinated was information on vaccine effectiveness (39.3%), death due to COVID-19 (37.7%), and recommendations from the Ministry of Health (36.1%). Most of the respondents (77.0%) were knowledgeable about vaccines, with half having high-perceived risks (52.5%) to COVID-19. While 55.7% and 52.5% had, high perceived barriers and benefits to COVID-19 vaccines respectively. The reasons for vaccine rejections included vaccine safety, indecisiveness, underlying medical conditions, herd immunity, non-transparent data, and use of traditional or complementary medicine.

**Conclusion:**

The study explored the multitude factors that drive perception, acceptance, and rejection. The qualitative approach with a small sample size provided more data point for interpretations and allowed participants to express themselves. This is important to develop strategies to create public awareness on vaccines not just for COVID-19 but any infectious diseases that can be curbed through vaccination.

## Introduction

Coronavirus disease 2019 (COVID-19) caused by the novel severe acute respiratory syndrome coronavirus 2 (SARS-CoV-2) was first reported in December 2019 in Wuhan, China and quickly spread to the rest of the world. The World Health Organization (WHO) announced COVID-19 as a pandemic on the 11^th^ March 2020 [1]. Malaysia reported the first case on 25^th^ January 2020 and traced it back to three Chinese nationals who were close contacts to a Singaporean patient [2]. The COVID-19 pandemic has triggered unprecedented public health concerns and increased the burden on healthcare systems worldwide [3]. As of March 2022, there have been approximately 452 million people infected with the COVID-19 worldwide, resulting in more than 6 million deaths [4].

To bring the pandemic to an end, governments worldwide implemented both biosafety and biosecurity measures such as lockdown, curfew, community quarantine and social distancing, sanitization and wearing of protective gears like masks. These measures managed to partially delay and prevent the spread of the virus, and flatten the curve [5, 6]. However, the implementation of lockdown particularly resulted in disruption in daily life that affected socioeconomic activities and generated social instability, and negatively impacted people’s mental health and well-being in many countries, including Malaysia [7, 8]. Additionally, lockdown was only able to contain the spread for a short period and the resumed economic sectors resulted in resurgence of cases and new wave of the pandemic [8].

The global COVID-19 vaccine administration approved by the US Food and Drug Administration (FDA) in December 2020 reported reduced number of new COVID-19 cases in 2021 and number of hospitalizations and deaths associated with the disease [9]. This situation resulted in accelerated use of COVID-19 vaccines in halting COVID-19 pandemic and restoring normal social and economic life [10]. In Malaysia, the National COVID-19 Immunization Program (PICK) that was initiated in February 2021 aimed to vaccinate 80% of Malaysians. The vaccines were given free to Malaysians and foreigners living in Malaysia who registered voluntarily. However, the government had to take extra measures in handling vaccine hesitancy. According to the WHO, vaccine hesitancy can be defined as ‘delay in accepting or refusing vaccination despite the availability of vaccination services’[11]. Several countries close to eliminating infectious diseases in the past such as measles and polio have seen a resurgence due to vaccine hesitance [12]. The main concern with COVID-19 vaccine hesitancy is that unvaccinated people can act as SARS-CoV-2 reservoirs. They could trigger a new epidemic or pandemic from an endemic infection of SARS-CoV-2 [13].

Hence, this study was designed to 1) evaluate self-assessed knowledge on COVID19 vaccines; 2) assess perceived risks and benefits of COVID-19 vaccines, barriers, and public perceptions towards PICK program; 3) address concerns of vaccine-hesitant public to increase public acceptance and develop public awareness strategies.

## Methods

### Study design and respondents

This cross-sectional online survey study was conducted between July 21 and August 25, 2021, using an embedded mixed-methods approach to explore the knowledge and perception of the COVID-19 vaccine among adult Malaysians aged 18 years and above who did not enroll for the COVID -19 vaccine. The qualitative component of the research was incorporated into a main cross-sectional study that focused on quantitative data. The qualitative section was intended to supplement the quantitative section by explaining why people were not enrolling for the COVID-19 vaccine and their recommendations for a National COVID-19 Immunization Program.

### Data collection tools and procedures

The data were obtained via an online questionnaire on the Goggle Forms platform. A reliability test was performed to assess the internal consistency of all the constructed items, which resulted in a Cronbach’s alpha of greater than 0.6. The research instrument for quantitative method was a 49-item questionnaire comprised of seven sections derived from Mohamed et al [14]:(1) Sociodemographic information, chronic illness history, and COVID-19 experience (10 items); (2) information sources and factors that influence the decision to be vaccinated (3 items); (3) knowledge about COVID-19 vaccines (7 items); (4) perceived risks associated with COVID-19 vaccines (3 items); (5) perceived barriers to COVID-19 vaccines (8 items); (6) perceived benefits associated with COVID-19 vaccination (6 items); and (7) perceptions regarding the ongoing National COVID-19 Immunization Program (5 items).

Following the embedded mixed-methods design, we developed two open-ended questions to qualitatively assess: (1) "Please state your reason why you have not registered or have no intention to register at all for COVID-19 vaccines?" and (2) "Please tell us if you have any suggestions for improvement about COVID-19 vaccine delivery". Data from respondents who were not willing to get vaccination were extracted from the overall data and further analyzed.

### Ethical consideration

The Ethics Committee of Universiti Sains Islam Malaysia approved this study, conducted under the code project of USIM/JKEP/2021-126. By willingly completing and submitting the questionnaire, the respondents agreed to participate in this study.

### Data management and analysis

For analysis, the data were coded and entered into SPSS-for-Windows version 23. Categorical variables were summarized using frequency and proportions, whereas continuous variables were summarized using mean and standard deviation. Correctly answered items were given 1 point each, and the overall COVID-19 vaccination knowledge score was calculated. Marks over the median score indicated a good degree of knowledge, while those below the median score indicated a low level of understanding. “Agree” or “Disagree” were the response options for all perceived and perception construct items. A higher perceived likelihood of an experience or construct was associated with a higher total response value. High perceived levels were defined as marks over the total median score, while low perceived levels were defined as marks below the total median score.

The Mann-Whitney test for two categorical variables and the Kruskal-Wallis test used for more than two categorical variables were utilized to determine the differences between groups for selected demographic variables (age group, gender, education, household income categories, and high-risk chronic illness condition) associated with total knowledge score. Thematic analysis was used to code and analyse the qualitative data after transcription and translation of the open-ended response. The data was analysed until the research team reached theme saturation. The qualitative study findings were used to supplement the quantitative data.

## Results

### Socio-demographic characteristics

In total, 3053 respondents completed the questionnaire, with 61 (2%) respondents answered not willing to be vaccinated. The mean age of the respondents was 34.28 years (SD = 10.30), ranging from 21–57 years. The average income of the respondents was RM 7,643.22 (SD = 11910.93). The details and analysis of the 61 respondents are presented. Table 1 summarizes their sociodemographic characteristics. More than half of the respondents were females (60.7%) and living in urban areas (62.3%). Most of the respondents reported having no known chronic medical illness (88.5%) and have never been diagnosed with COVID-19 (86.9%).

**Table 1:** Socio-demographic characteristics of respondents (*n* = 61).

Characteristic		*n*	%
Age group	21–39	40	65.6
	40–57	21	34.4
Gender	Male	24	39.3
	Female	37	60.7
Current residence ^a^	Sarawak, Sabah & WP Labuan	3	4.9
	Northern Zone	15	24.6
	Middle Zone	15	24.6
	Southern Zone	19	31.1
	East Coast Zone	9	14.8
Locality	Urban	38	62.3
	Rural	23	37.7
Education	No formal education	1	1.6
	Primary	1	1.6
	Secondary	10	16.4
	Diploma	17	27.9
	Degree	24	39.3
	Master/PhD	8	13.1
Occupation category ^b^	Unemployed, students, retiree	26	42.6
	Manager & Professionals	21	34.4
	Technicians & Associated Professionals	9	14.8
	Clerical support workers & Basic workers	4	6.6
	Services & Sales Employees	1	1.6
Income category ^c^	Low income: Less than RM 4850	25	42.4
	Middle income: RM4850-RM10970	24	40.7
	High income: Above RM 10971	10	16.9
	Missing data	2	
With at least 1 chronic illness	Yes	7	11.5
	No	54	88.5
Diagnosed with COVID-19	Yes	2	3.3
	No	53	86.9
	Not sure	6	9.8

^a^ Northern Zone: Kedah, Perak, Pulau Pinang, Perlis; Middle Zone: Selangor, WP Kuala Lumpur, WP Putrajaya; Southern Zone: Negeri Sembilan, Melaka, Johor; East Coast Zone: Kelantan, Terengganu, Pahang

^b^ Malaysia Standard Classification of Occupations (MASCO) 2020

^c^ Department of Statistics Malaysia (DOSM), 2020

### Information sources and trust

Most respondents acquired information regarding the COVID-19 vaccine through social media, i.e. Facebook, Instagram, YouTube, and Twitter (73.8%), Ministry of Health (MOH) Malaysia’s official website (65.6%), online news portal (63.9%) and instant messaging platform, i.e. WhatsApp, Telegram, and SMS (62.3%). Only 37.7% of the respondents acquired information from medical or healthcare personnel. Even so, the most trusted source by respondents for COVID-19 vaccines information were doctors (62.3%) and scientists (52.5%). Only 19.7% and 18.0% of the respondents had trust in the information provided by the government and the WHO respectively. The other option found that some of the study respondents stated that the information was not transparent.

Respondents reported the factors that influence the decision to be vaccinated were the effectiveness (39.3%), the number of reported deaths due to COVID-19 (37.7%), recommendation/s from MOH (36.1%) and assurance that the vaccine is halal (32.8%).

### Knowledge of COVID-19 vaccines

**Fig 1:**
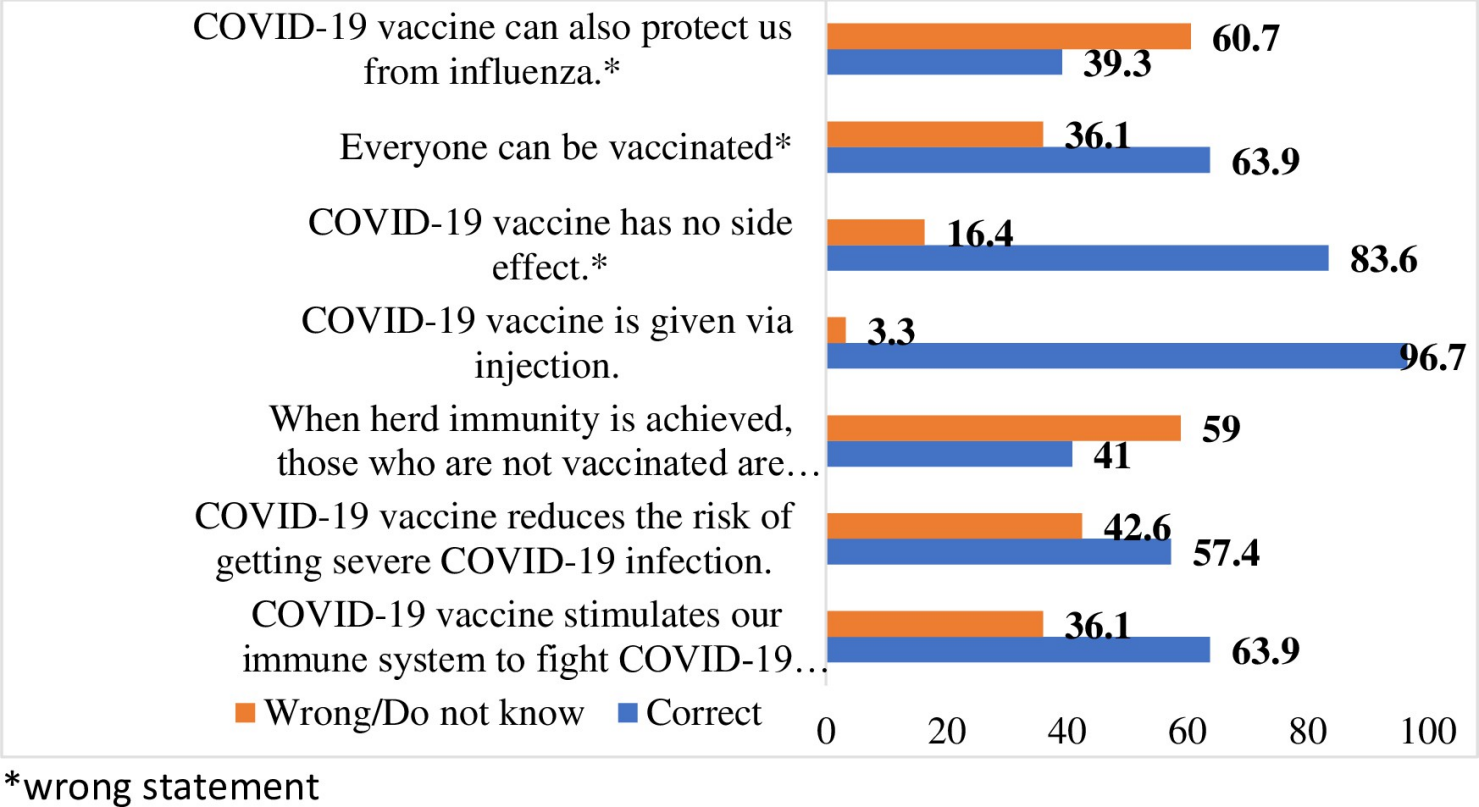
Knowledge item about COVID-19 vaccine (*n* = 61).

The average knowledge score was 4.46 (SD = 1.311), with 23.0% (n = 14) exhibiting a poor level of knowledge. Only about half of respondents knew that the COVID-19 vaccine could minimize the risk of long COVID-19 and its severity (57.4%). Meanwhile, less than half of the respondents knew that those not vaccinated are protected when herd immunity is achieved (41.0%) [Fig 1]. Differences in knowledge scores among different demographic characteristics were assessed using inferential analysis. The results show that none of the demographic characteristics differed significantly for the knowledge score [Table 2].

**Table 2:** Demographic characteristics of respondents and knowledge score (*n* = 61).

Variables		No. (n)	Mean (SD)	Median (IQR)	P-value
Age group	21–39	40 (65.6)	4.35 (1.41)	4 (2)	0.440
	40–57	21 (34.4)	4.67 (1.11)	5 (2)	
Gender	Male	24 (39.3)	4.46 (1.02)	4.5 (1)	0.921
	Female	37 (60.7)	4.46 (1.02)	4 (2)	
Locality	Urban	38 (62.3)	4.39 (1.41)	4 (2)	0.613
	Rural	23 (37.7)	4.57 (1.16)	5 (1)	
Education category	Lower Education	12 (19.7)	4.67 (1.92)	5 (2)	0.318
	Higher Education	49 (80.3)	4.41 (1.14)	4 (2)	
Income category	Low income	25 (41.0)	4.28 (1.02)	4 (2)	0.453
	Medium income	24 (39.3)	4.75 (1.30)	5 (2)	
	High income	10 (16.4)	4.10 (1.729)	4 (2)	
Chronic diseases	Yes	54 (88.5)	4.43 (1.13)	5 (2)	0.935
	No	7 (11.5)	4.46 (1.34)	4(1)	

### Perceived risk of COVID-19

The mean perceived risk score was 1.51 (SD = 1.30), with 52.5% of the respondents having high perceived risk regarding COVID-19. More than half of the respondents agree that they are at risk of getting infected (59.0%) and can transmit the virus to others (55.7%). However, most of the respondents disagree that they are at a higher risk of getting severe COVID-19 infection (63.9%).

### Perceived barriers to get vaccinated

**Fig 2:**
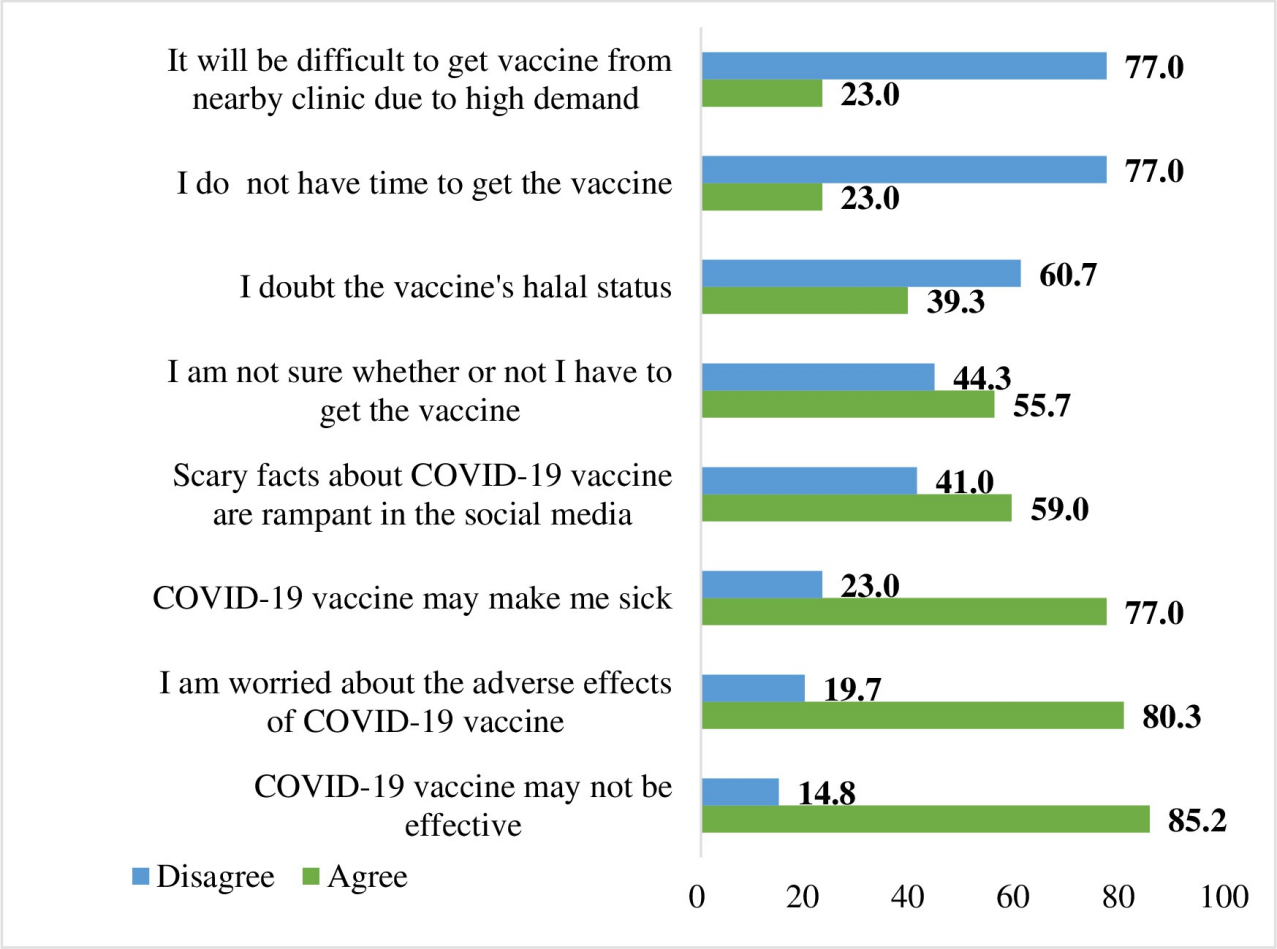
Perceived barriers to be vaccinated (*n* = 61).

Perceived barriers are factors that influence the rejection to be vaccinated. The average perceived barriers score was 3.75 (SD = 1.48), with 44.3% exhibiting low level of perceived barriers. Many of the respondents believed that the COVID-19 vaccines are ineffective (85.2%), has negative adverse effects (80.3%) and can make them sick (77.0%). Meanwhile, 59.0% of respondents agree that the scaremongering about the COVID-19 vaccines was the contributing factor to reject the vaccines [Fig 2].

### Perceived benefits

The mean of the perceived benefits that affect the respondent’s decision to be vaccinated was 3.30 (SD = 2.27), with only slightly more than half the respondents having high perceived benefits from the COVID-19 vaccines (52.5%). Less than half of respondents think that the media coverage of vaccines is clear (44.3%), that vaccines are safe (44.3%) and that vaccines can help them return to normal life again (42.6%). Nevertheless, 73.8% of responders said they will accept vaccines if doctors recommend them.

### Perceptions regarding COVID-19 vaccine administration

The mean perception score was 3.08 (SD = 1.05), with only 32.8% of the respondents having negative perceptions regarding the COVID-19 vaccination program. Most respondents agree that every Malaysian has the right to accept or reject vaccination (85.2%). While only 26.2% of respondents agree that the government should make the COVID-19 vaccine compulsory for all Malaysian citizens, and 37.7% felt that the information given by MOH is adequate.

### Reasons behind vaccine hesitancy

A total of 43 respondents explained their reasons for rejecting COVID-19 vaccines. Through their qualitative response, our data were distilled into seven main themes: (1) vaccination concern, (2) still studying the best option, (3) medical reasons, (4) expecting herd immunity from others, (5) lack of trust in data transparency, (6) no guardian consent and (7) opting to use traditional/complementary medicines. The illustrative quotes of emergent themes are presented in Table 3. We describe each of these emergent themes below.

**Table 3:** Analysis of themes for the reasons resulting in vaccine hesitancy.

List of themes	Illustrative quotes
1 a) Concerns about the vaccine: Under clinical trial	*“This vaccine is still in the trial stage*. *The health minister confirmed it in parliament*. *I don’t want to be a laboratory rat because many have had harmful effects after being vaccinated”* [49 years old, female]
	*“I am not anti-vaccine because I am a recipient of HPV*, *Rubella*, *BCG*, *etc*. *I am only COVID-19 anti-vaccine that is STILL IN the TRIAL PHASE*. *I will take it after that phase ends in 2023”* [41 years old, female]
1 b) Side effects	*“Long-term side effects are still unknown*. *The effects on natural immune responses have yet to be detected*. *Many things are still uncertain about the Covid-19 vaccine*, *but its use is widely forced and is of great concern”* [36 years old, male]
1 c) Unsafe and ineffective	*“In the self-informed consent form*, *it states that there is no guarantee the vaccine will protect against Covid-19*.*”* [42 years old, male]
	*“Not confident in the long-term efficacy and safety of COVID vaccines”* [43 years old, male]
2) Still studying the best option	*“I am still studying the best options for myself”* [36 years old, female]
3) Medical reason	*“Had allergies to medications*, *food and environmental changes*, *had been hospitalised for strong allergies*. *Thus*, *less confident and do not want to take risks on existing disease”* [25 years old, male]
4) Herd immunity from others	*“To save the government money because of herd immunity from people around my environment who have already taken the vaccine”* [43 years old, male]
5) Data is not transparent	*“News from abroad shows a variety of severe side effects or deaths to vaccine recipients*. *However*, *the cases have been silenced by hidden agenda (money talks)”* [41 years old, female]
	“*Data is not transparent*.” [44 years old, female]
6) No guardian consent	*“Didn’t get permission from my family”* [23 years old, female]
**7)** Traditional/ complementary medicine used	*“I practice natural remedies in preventing disease”* [42 years old, male]
	*“For now*, *take supplements and Sunnah foods to strengthen immunity*, *such as Vitamin C and other supplements such as B-complex*, *multivitamins to boost the immune system”* [49 years old, male]

### Suggestions for improvement of the COVID-19 vaccine programme

Twenty-four participants responded to these open-ended questions. The suggestions were categorized into six themes as follows: (1) Stop the vaccination program, (2) no mandatory vaccination, (3) no changing in law to force vaccination, (4) education on adverse events following immunization (AEFI), (5) data transparency on AEFI and (6) vaccination-related conspiracy theory. Table 4 contains illustrative quotations from the qualitative response on emerging themes.

**Table 4:** Analysis of themes for the suggestion on the *COVID-19 vaccine programme*.

List of themes	Illustrative quotes
1) Stop the vaccination Program	*“Stop vaccination in MALAYSIA*.*”* [42 years old, male]
2) No mandatory vaccination	*“Vaccination is not mandatory for individuals*. *Medical apartheid*. *One size does not fit all”* [42 years old, male]
	*“Stop the mandatory call because the right as a citizen is free to choose”* [44 years old, female]
3)No changing in law to force vaccination	*“Don’t change the law to force vaccination*, *like if we want to change the name for the land ownership*, *dine in a restaurant*, *buy something in the supermarket*, *perform Solah in Masjid*, *or any other religious place”* [40 years old, male]
	*“Vaccination is voluntary*, *and there is no need to discriminate against people who do not receive the vaccine based on “safety” factors”* [49 years old, male]
4) Education on AEFI	*“The government should better educate the people about vaccines—post-vaccine effects*.*”* [36 years old, female]
5) Data transparency on AEFI	*“The government needs to be transparent in disseminating the vaccine’s side effects and the data that Covid-19 patients who took the vaccine still got severe side effects*, *and some even died*.*”* [32 years old, female]
6) Vaccination-related conspiracy theory	*“Not a suggestion but the fact that this vaccine is related to the agenda of depopulating human beings*.* *.* *. *I don’t believe in infidels*.* *.* *.*”* [38 years old, male]
	*“Later only*. *This vaccine is just a political game*. *Imagine if everyone in Malaysia is vaccinated*, *we are still exposed to Covid-19*, *and there are still positive ones*. *If we talk about wanting to reduce how sick Covid-19 is*, *please understand*, *it’s still positive*, *still there too*. *What’s the point*? *The business of big people is like this*. *Malaysia is just a puppet”* [38 years old, male]

## Discussion

As of 15 March 2022, 47.4% of Malaysian population has received the booster dose, and 79.6% and 84.5% are fully vaccinated with two doses and have received the first dose respectively [15]. Despite the high rate of vaccination, there are individuals who are still reluctant to register in the PICK program and those who are afraid to get heterologous booster dose. Respondents who did not register to be vaccinated provided very useful information on their risk perception, trusted sources, knowledge level on vaccines and reasons for their hesitancy. Information in this area is crucial to understand the factors driving vaccine hesitancy.

A global systematic review on COVID-19 vaccine acceptance has shown that hesitancy seems to be increasing even with mass vaccination campaigns, particularly in the working-age population [16, 17]. Our result is also in line with the survey among vaccine-hesitant individuals by Schwarzinger et al. [18], which found high numbers of respondents in the younger age group, female gender, and urban residents.

Results from this study indicate that most respondents received information regarding COVID-19 vaccines through social media, as reported in other studies conducted in Malaysia [14, 19]. The use of social media has its advantages and disadvantages in disseminating information, with studies showing that those who received their information through social media are more likely to be vaccine hesitant than those who received their information mainly from conventional or mainstream media [20, 21]. Majority of the population rely on social media for news and information on COVID-19 vaccine, and this is linked to a lack of implicit confidence on the government and health professionals, and strong belief in various conspiracy theories and doubts. This finding is justified by the quantitative and qualitative components of our results. Only a small number of respondents had trust in the government and the WHO. At the same time, some questioned the data transparency provided by both the government and the WHO.

In our study, respondents’ knowledge of COVID-19 vaccines was good, which was higher than the previous study conducted among general Malaysian populations in December 2020 before the PICK program commenced [14]. Before the launch of the PICK program, the government through MOH has been continuously promoting the benefits of vaccination through various communication and media channels [22, 23]. Nevertheless, only less than half of the respondents knew the importance of herd immunity. In the battle to end COVID-19, rapid and widespread vaccination is critical in achieving herd immunity and this can be delayed if the population does not understand its importance and ways to achieve it.

Only half of the respondents had high perceived risks of COVID-19 infection. Although many people were concerned about the possibility of contracting COVID-19, only a small number believed they were at increased risk of contracting severe illness and transmitting the virus to others. This indicates the need to improve risk perception of COVID-19 among all the populations, as high-risk perception translates into an important determinant predicting vaccines uptake in many infectious disease outbreaks and has been found to enhance epidemic control [24]. The low- risk perception could also be due to COVID-19 being a novel disease and little was known to the public at the start of the pandemic. On the other hand, vaccines, especially the mRNA and mass adult vaccination are new to the masses, creating fear of the unknown [25]. This again calls for a strong public awareness campaign.

The minor barriers to receiving the vaccine are time, accessibility, and halal status. In response to the ongoing COVID-19 outbreak, Malaysia has increased immunization rates by opening numerous vaccination facilities until 9 p.m. on weekends and public holidays [26]. Meanwhile, the Malaysian National Fatwa Committee announced that the COVID-19 vaccine is permissible after thorough evaluation of its technology and ingredients [27, 28]. This helped shift public perception, especially among the Muslim population and increased the acceptance towards the vaccines at the late stage of the pandemic.

The most common barrier among the respondents were the effectiveness of the vaccines, side effects and scary news or misinformation on social media. This finding was supported by the qualitative result, where most of the respondents quoted the negative information and conspiracy theory as the reason for vaccine hesitancy. These barriers are also the most cited factors influencing vaccine risk perceptions and acceptance of vaccines against COVID-19 and other infectious diseases among global population [29, 30]. Thus, acknowledging public fears and negative emotions while clearing their misinformation and conspiracy theories may help to increase vaccine confidence.

Meanwhile, only half of those surveyed thought the COVID-19 vaccination provided high benefits. Even though most respondents will take vaccines if their doctors recommend them, factors such as vaccine safety and unclear information about vaccines in the media restrict public understanding on the benefits of vaccines. One of the respondents suggested that the government should stop the vaccination effort. Many also cited that negative information about COVID-19 vaccines such as side effects and conspiracy theories outweigh the benefits of vaccination. Greater concerns about the perceived risks associated with vaccine safety versus the perceived severity of the COVID-19 is shown to contribute to vaccine rejection in other studies among participants who are hesitant in getting vaccinated [31, 32]. Thus, eliminating the barriers to vaccine acceptance is crucial for the public to see the benefits of vaccination through an evidence-based lens.

On a positive note, most participants had good perception of the PICK programme. Nevertheless, for many of them, the information on the vaccines given by the MOH was still inadequate. Respondents inquired the hidden agenda and data transparency, including AEFI, which impacted hesitation in the qualitative findings. The capacity to conduct post-licensure vaccine safety surveillance varies between nations, notably between low- and middle-income countries [33, 34]. Hence, to gain public trust, the Malaysian government should try to disclose AEFI data at a regular interval with transparency and, if possible, actively disseminate data on post-vaccination surveillance.

Finally, there are concerns about freedom of choice among vaccine hesitant. COVID-19 vaccination is vital, according to Dhama et al. [35], and should be mandatory for all, regardless of community or county. Nonetheless, COVID-19 vaccination is not legally mandatory in Malaysia [14]. However, employers such as the federal government, healthcare, and educational institutions, have made vaccination compulsory for all employees [36]. As a result, respondents expressed anxiety about government-enforced laws restricting their movement in public places and fears being forced to be vaccinated by the government through legislation. This is consistent with earlier studies in which vaccine-hesitant persons want the right to choose whether to get vaccinated, and they do not wish their vaccination status tied to their freedom to move around [31, 37].

This study is among the first to assess the knowledge and perception of COVID-19 vaccines, focusing on Malaysian who are reluctant to register for the COVID-19 vaccine program. We employed a mixed-methods design to understand the perceptions and reason for the COVID-19 vaccine-hesitant. As this data was collected online, there may be low representation from vulnerable individuals and from rural areas who do not have access to the internet or have limited internet access. Finally, since the disease is still spreading at the time of the research, rapidly shifting knowledge and perception about COVID-19 vaccinations may impact the relevance of the findings at the later stage of the pandemic.

Vaccination rates must be high and rapid to effectively control vaccine-preventable illnesses. The vaccine hesitant problem is multifaceted, requiring responses from both individuals and system. Vaccine-hesitant individuals may influence and transmit negative perceptions, potentially affecting community vaccination delays or refusals. Thus, addressing vaccine-hesitant individuals’ concerns is crucial to promote vaccine confidence. Our research reveals that many respondents trust healthcare experts for vaccine information and will get vaccinated if they recommend it. Hence, healthcare providers must educate and raise confidence on vaccination and the importance of achieving herd immunity. The government must also monitor social media for misinformation or "fake news" influencing vaccine decisions and promoting vaccine confidence by tailoring information to address concerns of the vaccine-hesitant individual. Malaysia has laws in place to charge those spreading fake news about the vaccines.

## Conclusions

A small proportion of Malaysian population have concerns about the COVID-19 vaccine and remain unwilling to get vaccinated even though it is available and provided for free. Even though their level of knowledge was reasonable, their low perceived risks of COVID-19, lack of perceived benefits from vaccination, high perceived risks of vaccination; and perceived barriers on the accessibility of the vaccines contributed to vaccine hesitation. In addition, several conspiracy theories and disbelief in data supplied by local and international health authorities were put forth to justify their stance. This was mainly due to the misconceptions disseminated through social media as a source of information about the vaccine. Acknowledging their barriers and concerns is essential to increase acceptance. Therefore, the community must be provided with more information on the safety and effectiveness of vaccines and the role vaccines play in achieving herd immunity. It is also important to ensure scientific communication is carried out continuously and not as a fire-fighting approach during time of crisis.
